# Editorial: Drug development for respiratory infectious diseases and related complications in other systems

**DOI:** 10.3389/fmed.2026.1800928

**Published:** 2026-03-03

**Authors:** Xueping Yu, Yu Wang, Alessandra Oliva, Mingshan Xue

**Affiliations:** 1 Department of Infectious Disease, Clinical Medical Research Center for Bacterial and Fungal Infectious Diseases of Fujian Province, Fujian Medical University Affiliated First Quanzhou Hospital, Quanzhou, Fujian, China; 2 Key Laboratory of Screening and Control of Infectious Diseases (Quanzhou Medical College), Fujian Provincial University, Quanzhou, Fujian, China; 3 Department of Cardiology, Shidong Hospital, Shanghai, China; 4 Department of Public Health and Infectious Diseases, Faculty of Pharmacy and Medicine, Sapienza University of Rome, Rome, Italy; 5 Department of Respiratory and Critical Care, Guangzhou Medical University, Guangzhou, China

**Keywords:** antiviral therapy, atypical pneumonia, drug development, real-world evidence, respiratory infectious diseases, RSV immunization

Respiratory infectious diseases remain one of the most time-sensitive arenas in clinical medicine. Pathogens evolve, transmission patterns shift, and outcomes are shaped not only by the organism, but also by host vulnerability, comorbidities, and the care pathway that determines who receives which intervention—and when. In this setting, drug development cannot be reduced to “one pathogen, one pill.” It must span prevention, early outpatient therapy, inpatient rescue strategies, and the management of complications in patients for whom standard approaches may be unsafe or insufficient.

This Research Topic was launched to reflect that full continuum. Across nine accepted contributions, three priorities recur: (i) aligning pathogen-directed therapy with real-world constraints (drug interactions, access, timing, and workflow); (ii) translating mechanistic signals into testable, deployable candidates and combinations; and (iii) anticipating complications beyond the lung—where neuromuscular disease, cancer therapy, and chronic airway disease can shift the risk–benefit balance.

## From antiviral candidates to real-world effectiveness: narrowing the “translation gap”

Translation starts with two deceptively simple questions: do candidate antivirals improve meaningful outcomes in practice, and can mechanistic screening be disciplined enough to yield a short list of testable leads. Chen J. et al. compare oral azvudine with nirmatrelvir/ritonavir in hospitalized COVID-19 patients and report broadly comparable effectiveness and safety in routine care. Importantly, their results read as a reminder that antiviral choice is often determined by context—drug–drug interactions, contraindications, availability, and the timing of presentation within an evolving inpatient pathway—rather than by the assumption of a single universally “best” option. Liu et al. approach the same translation challenge from the opposite direction. By integrating *in silico* screening with bioactivity validation, they identify candidate anti-SARS-CoV-2 and anti-inflammatory constituents within Qingyan Dropping Pills, showing a practical way to triage multi-component therapies without treating them as a black box. Together, these papers sketch a workable “two-lane road”: comparative effectiveness to test clinical utility on one side, and mechanism-to-candidate selection on the other. This perspective becomes even more consequential when pneumonia is atypical or severe—settings where the right drug is only available once the right diagnosis is made.

## Severe and atypical pneumonias: when diagnostics and timing matter as much as drug choice

Atypical and zoonotic pneumonias can deteriorate quickly, and diagnostic delay can erase the benefit of any antimicrobial. Nie et al. illustrate this challenge in a case report of severe Q fever pneumonia presenting during an influenza epidemic. Their report highlights how syndromic overlap can obscure a treatable etiology, and how molecular confirmation—particularly sequencing—can reopen the door to targeted therapy. Seen through a development lens, the message is clear: diagnostics are not ancillary to anti-infective evaluation; they define eligibility and the therapeutic window. Feng et al. extend this real-world framing to the ICU, describing severe Chlamydia psittaci pneumonia requiring invasive mechanical ventilation and summarizing experience with fluoroquinolone-based management. While prospective comparisons will be needed to refine regimen ranking, well-characterized ICU cohorts help clarify timing, safety signals, and endpoints that future trials can build on. From here, the Topic moves to pediatrics—where the translation gap is often less about identifying an active intervention and more about delivering it reliably and equitably.

## Pediatrics and prevention: pairing near-term tools with implementation reality

In children, supportive regimens and prevention strategies can have outsized downstream impact, but only if they are practical and scalable. Zeng et al. synthesize a large trial landscape to compare nebulized adjunct drugs added to azithromycin for non-severe pediatric Mycoplasma pneumoniae pneumonia via a network meta-analysis. By ranking commonly used inhaled add-ons, they push adjunctive care away from habit toward evidence-graded selection, while also revealing where heterogeneity in outcomes and protocols limits certainty—an actionable signal for standardization in future pediatric studies. At the prevention end of the continuum, Tate et al. provide an implementation framework for planning an RSV immunization program for infants using a long-acting monoclonal antibody. Their emphasis on delivery details—stakeholder coordination, logistics, surveillance, and equity—highlights a recurring lesson in respiratory infection control: population impact depends as much on implementation as on pharmacology. Taken together, these pediatric contributions bridge naturally to the Topic's final theme: how comorbidity and immune vulnerability outside the lung reshape both therapeutic choice and outcome.

## Complications beyond the lung: drug–disease interactions in vulnerable hosts

Respiratory infections often unfold in patients for whom guideline algorithms are least reliable, and in whom adverse effects can be as dangerous as the infection itself. Chen X. et al. review myasthenia gravis complicated by community-acquired pneumonia and lay out the high-stakes trade-offs clinicians face: some antimicrobials and supportive agents may worsen neuromuscular transmission, while changes in immunosuppression can trigger disease flare or leave infection uncontrolled. By making these competing risks explicit, the review offers a practical decision structure that can reduce avoidable harm in routine care. Beyond neuromuscular disease, immunosuppression and tissue injury after cancer therapy can create a different set of vulnerabilities. Tian et al. report Streptococcus dysgalactiae subsp. dysgalactiae bloodstream infection in breast cancer patients after radiotherapy and chemotherapy, underscoring that progression is sometimes driven not by pulmonary pathology alone but by invasive bacterial disease. Their paper points to a frequently overlooked development priority: drug strategy should be paired with surveillance and rapid diagnostics that enable timely escalation before systemic deterioration. Finally, prevention remains central in chronic airway disease. Shuai et al. evaluate non-typeable Haemophilus influenzae and *Moraxella catarrhalis* vaccine strategies in COPD through a systematic review and meta-analysis. Even when candidate vaccines show acceptable safety, demonstrating consistent reductions in exacerbations and hard outcomes is challenging—yet essential in the face of resistance pressures and the cumulative burden of recurrent infection. By clarifying where evidence is strongest and where gaps remain, their work helps define more rigorous next-step trial designs (population selection, endpoints, immunologic correlates, and regimen standardization).

## Outlook: toward platform thinking in respiratory anti-infective development

**Figure 1:**
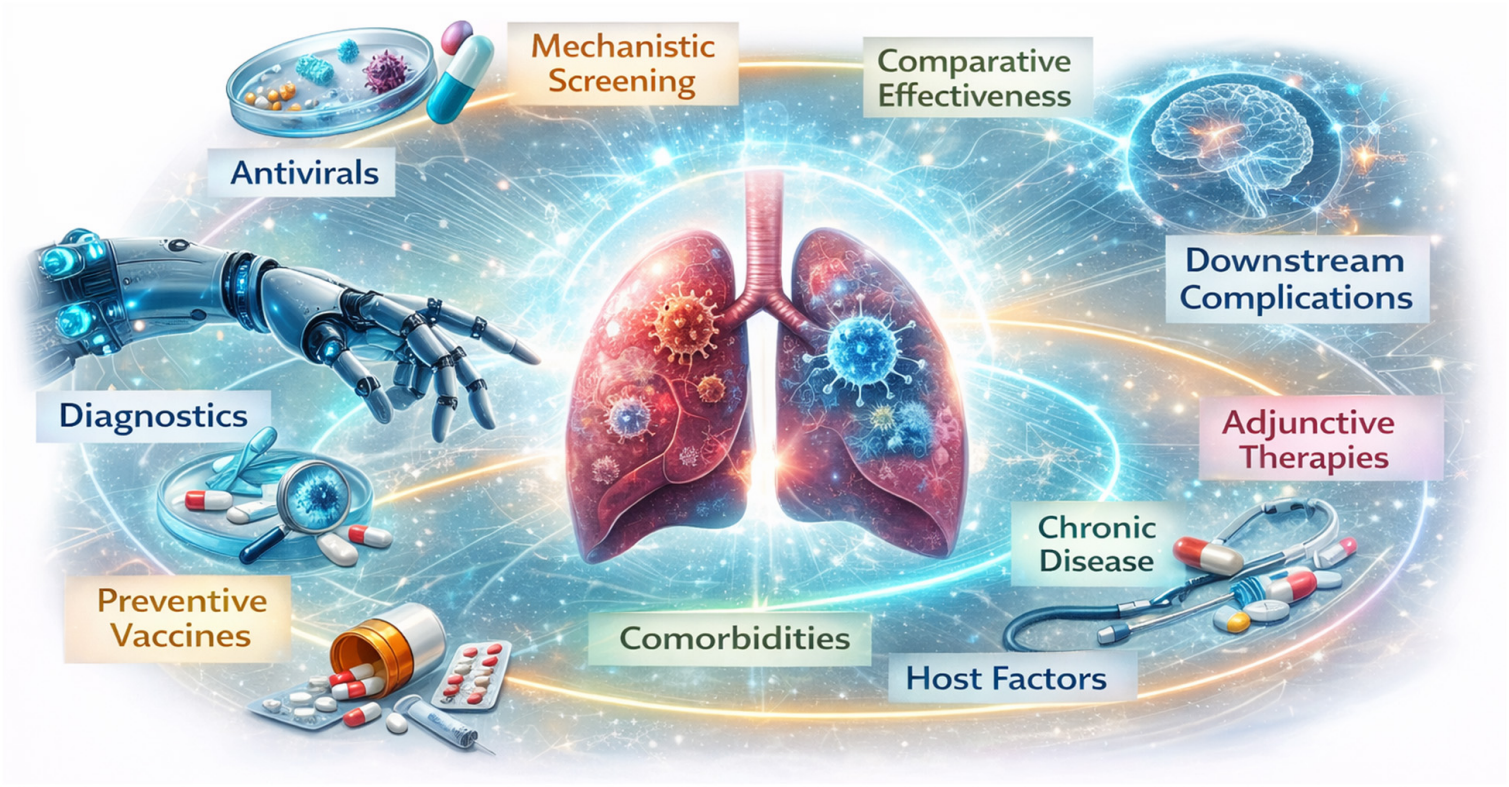
Translational roadmap for drug development in respiratory infectious diseases and related complications in other systems.

Across these nine papers, the take-home is less a table of contents than a development pathway (Figure 1). Progress is most likely when diagnostics and stratification are embedded into evaluation—particularly for atypical pathogens—so that therapies are tested in the patients and time windows where benefit is plausible. Comparative effectiveness studies remain essential to confirm whether efficacy translates into routine care once access, contraindications, and workflow constraints are accounted for. Implementation planning should be treated as part of the intervention, especially for prevention tools that only deliver impact when coverage is achieved.

Perhaps most importantly, the Research Topic reminds us that “respiratory infection” is often a systems problem: neuromuscular disease, cancer therapy, and chronic lung disease can change both susceptibility and tolerability, shifting the net benefit of any given strategy. Future work that combines structured diagnostics, standardized endpoints, and deliberate focus on high-risk subgroups will be best positioned to narrow the translation gap and improve outcomes across the full continuum of care.

